# The cost-effectiveness of iruplinalkib versus alectinib in anaplastic lymphoma kinase-positive crizotinib-resistant advanced non-small-cell lung cancer patients in China

**DOI:** 10.3389/fpubh.2024.1333487

**Published:** 2024-04-15

**Authors:** Zhanjing Dai, Jiayi Xu, Feng Chang, Wanxin Zhou, Ting Ren, Jiaxin Qiu, Yun Lu, Yuqiong Lu

**Affiliations:** School of International Pharmaceutical Business, China Pharmaceutical University, Nanjing, China

**Keywords:** iruplinalkib, alectinib, cost-effectiveness, anaplastic lymphoma kinase, non-small cell lung cancer, China

## Abstract

**Background:**

Iruplinalkib is a second-generation anaplastic lymphoma kinase (ALK) tyrosine kinase inhibitor (TKI) with efficacy in patients with ALK-positive crizotinib-resistant advanced non-small cell lung cancer (NSCLC), which is independently developed by a Chinese pharmaceutical company. This study examined the cost-effectiveness of iruplinalkib versus alectinib in the Chinese healthcare setting.

**Methods:**

A partitioned survival model was developed to project the economic and health outcomes. Efficacy was derived using unanchored matching-adjusted indirect comparison (MAIC). Cost and utility values were obtained from the literature and experts’ opinions. Deterministic and probabilistic sensitivity analyses (PSA) were carried out to evaluate the model’s robustness.

**Results:**

Treatment with iruplinalkib versus alectinib resulted in a gain of 0.843 quality-adjusted life years (QALYs) with incremental costs of $20,493.27, resulting in an incremental cost-effectiveness ratio (ICER) of $24,313.95/QALY. Parameters related to relative efficacy and drug costs were the main drivers of the model outcomes. From the PSA, iruplinalkib had a 90% probability of being cost-effective at a willingness-to-pay threshold of $37,863.56/QALY.

**Conclusion:**

Compared to alectinib, iruplinalkib is a cost-effective therapy for patients with ALK-positive crizotinib-resistant advanced NSCLC.

## 1 Introduction

Lung cancer has become a disease with the highest mortality worldwide (1). According to the latest statistics released by the Chinese National Cancer Center, the age-standardized incidence of lung cancer by the World Standard Population was 36.46/10^5^ and the age-standardized mortality by the World Standard Population was 28.09/10^5^ in 2016, both rank first (2). Non-small cell lung cancer (NSCLC) accounts for approximately 80–85% of lung cancer (3, 4), and approximately 5% of NSCLC tumors harbor anaplastic lymphoma kinase (ALK) gene rearrangements (5, 6). The 5-year survival rate for patients with late-stage (i.e., III/IV) NSCLC remains poor (7, 8). In addition, one study estimated that the 5-year direct medical expenditure for lung cancer was 31,248 United States dollars (USD) per patient in China in 2017. The total economic burden of lung cancer was estimated to be 25,069 million USD (0.121% of gross domestic productivity, GDP), and it is projected to increase to 53.4 billion USD in 2030 (9).

With the advancement in our knowledge of lung cancer and targeted therapies, there is some new improvement in patient outcomes. ALK-tyrosine kinase inhibitors (TKI) have demonstrated clinical improvements in both the progression-free survival (PFS) and the objective response rate (ORR) (10). Many ALK-TKIs have been recommended by the NSCLC clinical guidelines, such as the National Comprehensive Cancer Network Clinical Practice Guidelines in Oncology (NCCN) (11), Guidelines of the Chinese Society of Clinical Oncology (CSCO) for NSCLC (6), and the European Society for Medical Oncology (ESMO) (12). Crizotinib as the first-generation ALK-TKI showed superiority over chemotherapy (13) in ALK-positive NSCLC. However, the resistance to crizotinib, its unsatisfactory PFS benefits, and limited control of brain metastases drove the development of second-generation ALK-TKIs (alectinib, ceritinib, brigatinib, ensartinib, and iruplinalkib), which have higher selectivity and central nervous system (CNS) penetration and addressed the issue of crizotinib-resistance effectively (14, 15).

Iruplinalkib (WX-0593) is a novel, highly selective oral TKI that targets ALK. Iruplinalkib had superior inhibitory activity against the ALK-resistant mutants, especially the ALK-G1202R mutants. It showed better inhibitory activity than other second-generation ALK-TKIs and was similar to lorlatinib (third-generation) (16). In June 2023, iruplinalkib (WX-0593) as the class 1 innovative drug was approved for the treatment of ALK-positive crizotinib-resistant advanced NSCLC patients (aged ≥18 years) in China based on the INTELLECT study (ClinicalTrials.gov NCT04641754) (17) and has been included in the updated National Reimbursement Drug List (NRDL, 2023). The INTELLECT study (18) was a single-arm, multicenter (41 hospitals), phase II clinical trial conducted in China and showed that iruplinalkib had favorable clinical efficacy and manageable safety profiles. The ORR of iruplinalkib was 69.9% (95% confidence interval [CI] 61.7–77.2%), and the median PFS was 19.8 months (95% CI 14.5-NE), as assessed by the independent review committee (IRC). The overall survival (OS) data were immature (18).

Currently, the economic impact and value of iruplinalkib have not been evaluated in the second-line setting. On the background of rising healthcare costs and limited medical resources, pharmacoeconomics is used more widely across the world, especially in China, to help control the growth of drug costs (19). According to recommendations of the comparator selection in the China Guidelines for Pharmacoeconomics Evaluation 2020 (20), alectinib was selected as a suitable comparator because of the same indication, extensive clinical use, and a high recommendation in the guidelines. Therefore, this study aimed to evaluate the cost-effectiveness of iruplinalkib versus alectinib in treating ALK-positive crizotinib-resistant advanced NSCLC patients from the perspective of China’s healthcare system.

## 2 Methods

### 2.1 Model overview

**Figure 1:**
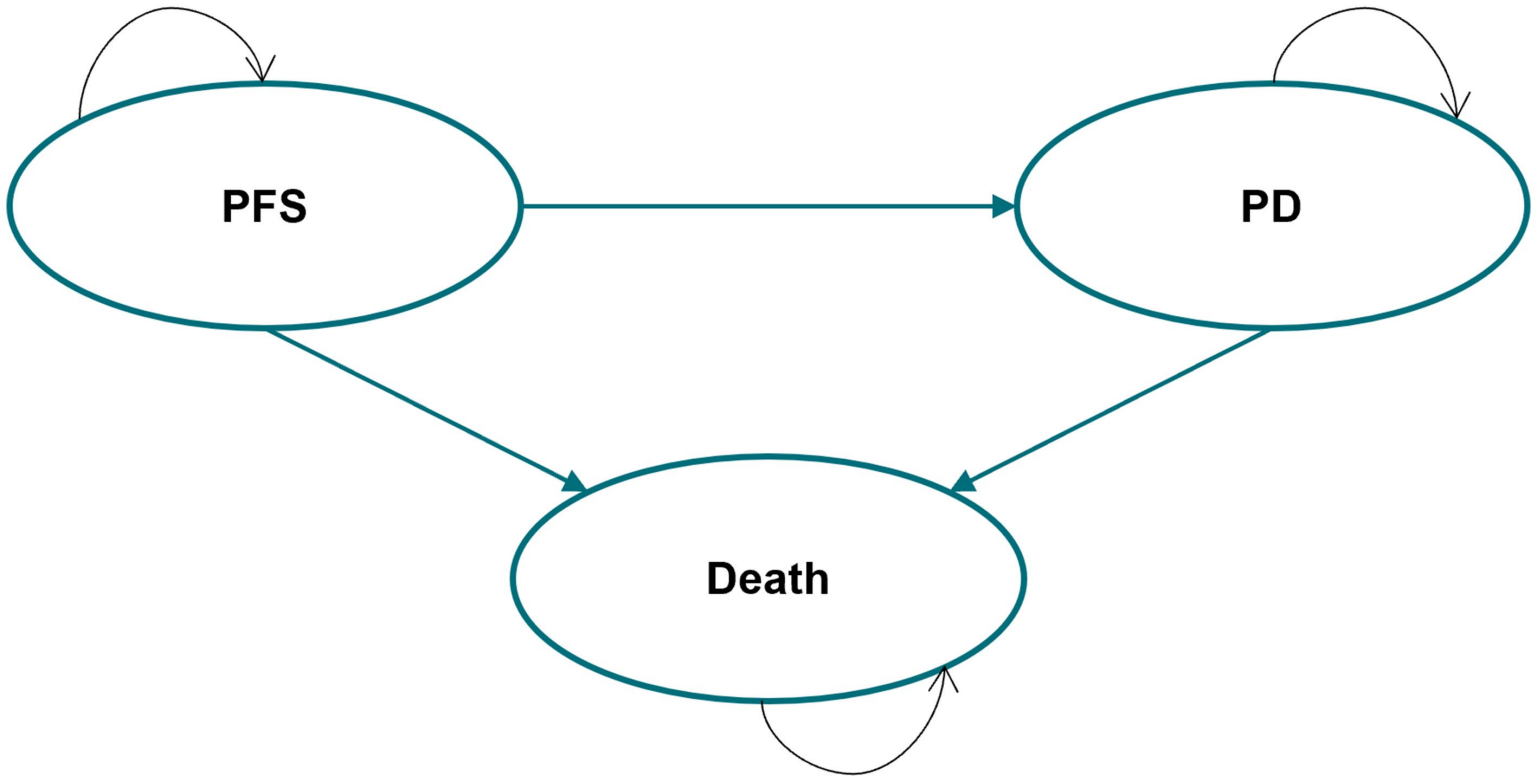
Partitioned survival model structure. PFS, progression-free survival; PD, progression disease.

This study adhered to the Consolidated Health Economic Evaluation Reporting Standards 2022 (CHEERS 2022) guidelines for health economic evaluation (21). A partitioned survival model (PSM) was developed in Microsoft Excel to evaluate the long-term benefits and costs of iruplinalkib and alectinib, as PSM was recommended by the National Institute for Health and Care Excellence (NICE) Technical Support Document TSD19 (22) and the China Guidelines for Pharmacoeconomics Evaluation 2020 (20). In addition, our prior systematic review (23) and another review (24) found that PSMs were the most widely used approach for cost-effectiveness analysis of second-line treatment in patients with ALK-positive advanced NSCLC. The model consisted of three health states, namely, progression-free survival (PFS), progression disease (PD), and death (Figure 1). The time horizon was 25 years, i.e., a lifetime perspective for these patients, since the mortality of both groups was approximately 90% (iruplinalkib) and 95% (alectinib). Moreover, the simulation starting age was 52 years according to the mean age in the trial of iruplinalkib, and living patients at the end of the simulation were close to the average life expectancy of the Chinese population in 2021 (78.2 years old). Costs and QALYs were discounted at 5% *per annum* in accordance with the China Guidelines for Pharmacoeconomics Evaluation 2020 (20). The cycle length in the base–case model was 3 weeks based on the administration cycle of iruplinalkib and subsequent therapy, and a half-cycle correction was applied to QALYs and all costs. Costs from previous years were adjusted using the consumer price index for healthcare of 2016–2023 and were shown in 2023 US dollars (1 USD = 7.08 CNY). For an intervention to be considered cost-effective, a willingness-to-pay (WTP) threshold of $37,863.56 per QALY (three times the GDP *per capita* of China) was used in the current analysis. This threshold is based on the China Guidelines for Pharmacoeconomics Evaluation 2020 (20), as well as published studies (25, 26, 27), which state that a treatment should be considered cost-effective if the ICER is between one and three times the GDP *per capita* of that country. The GDP *per capita* in China was. Additionally, a treatment is considered highly cost-effective if the ICER is less than one times the GDP *per capita*.estimated at $12,621.19 (￥89,358), which was taken from the Statistical Communiqué of the People’s Republic of China on the 2023 National Economic and Social Development (28).

### 2.2 Model inputs

#### 2.2.1 Efficacy data

Efficacy data (PFS and OS) for iruplinalkib were taken from the single-arm INTELLECT trial (18) carried out by the Qilu pharmaceutical company, which also provided the individual patient data (IPD) for this economic evaluation. Eligible patients who had ALK-positive crizotinib-resistant advanced NSCLC were enrolled. Efficacy data for alectinib were obtained from the ALUR trial (versus chemotherapy) (29) selected based on the same eligible patients, which was the latest phase 3 trial for alectinib in second-line treatment.

Due to the absence of head-to-head studies comparing iruplinalkib with alectinib, indirect comparisons were required in this study. Given the availability of IPD of iruplinalkib and aggregate data (AgD) of alectinib, the unanchored matching-adjusted indirect comparison (MAIC) (30) method was used since there was no common comparator in INTELLECT and ALUR. In terms of the matching variable selection, we referred to the published literature which used MAIC, Cox analysis results, clinical opinions, and availability of baseline characteristics. In addition, the recommendations in NICE guidance stated that all prognostic factors and treatment effect modifiers needed to be included in the unanchored MAIC (30). Finally, four matching variables, namely, age, sex, Eastern Cooperative Oncology Group (ECOG) performance status (PS), and CNS metastasis at baseline included Cox analysis results, and the reasons for the selection of matching variables are shown in Supplementary Tables S1, S2, respectively. The baseline characteristics before and after matching are shown in Table 1. The effective sample size (ESS) was 96.85. The IPD of iruplinalkib versus alectinib was required to calculate the hazard ratio (HR) based on the weights from MAIC. The IPD of iruplinalkib was provided by Qilu, while the IPD of alectinib was not available. Instead, KM graphs were digitized using GetData Graph Digitizer to create pseudo-IPD using the algorithm by Guyot et al. (31). After MAIC adjustment, PFS-HR of iruplinalkib versus alectinib was 0.580 (95% CI 0.383–0.878) and OS-HR was 0.746 (95% CI 0.471–1.184).

**Table 1:** Baseline characteristics before and after MAIC adjustment.

Matching variables	Iruplinalkib (INTELLECT)		Alectinib (ALUR)
	Before	After	
N/ESS	*N* = 146	ESS = 96.85	*N* = 72
Age [Mean (SD)]	52.4 (10.4)	54.6 (13.0)	54.6 (13.0)
Male(%)	47.3%	58.2%	58.2%
ECOG PS 0/1(%)	96.6%	92.4%	92.4%
CNS metastasis at baseline(%)	61.6%	62.0%	62.0%

ESS, effective sample size; SD, standard deviation; ECOG PS, Eastern Cooperative Oncology Group performance status; CNS, central nervous system; N, number of patients.

**Figure 2:**
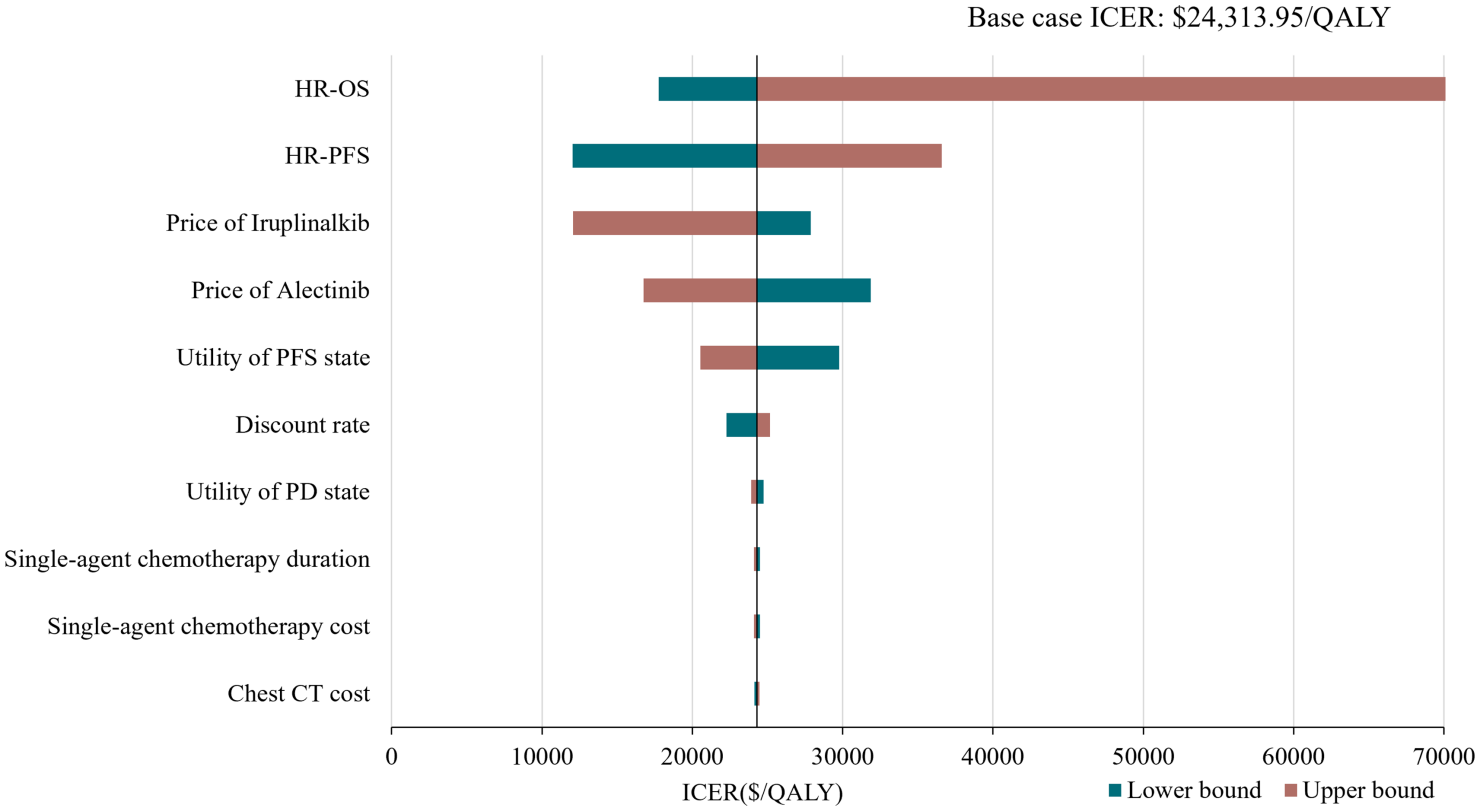
Tornado diagram of deterministic sensitivity analysis. HR, hazard ratio; PFS, progression-free survival; OS, overaal survival; ICER, incremental cost-effectiveness ratio.

Six commonly used parametric survival models, namely, exponential, Weibull, Gompertz, log-logistic, lognormal, and generalized gamma, were recommended by NICE guidance (32) and were fit for PFS and OS of iruplinalkib based on the IPD. On the basis of clinical rationality, visual fit, and statistical goodness-of-fit [Akaike information criterion (AIC) and Bayesian information criterion (BIC)], the log-normal distribution was selected for PFS and the generalized gamma distribution was selected for OS (AIC and BIC for PFS and OS are shown in Supplementary Table S3, the parametric survival curve fits are shown in Figures 1, 2). The HRs calculated based on the MAIC were then applied to adjusting the PFS and OS curves for alectinib. In addition, age-specific mortality was also applied when performing the cohort analysis to accurately simulate the survival status of patients. The age-specific mortality was driven by the China Population Census Yearbook 2020 (33).

#### 2.2.2 Utility inputs

Utility values of PFS and PD were derived from a study of health state utilities in NSCLC conducted by Nafees et al. (34), as trials of the two interventions did not measure patients’ quality of life. Nafees B et al. used the time trade-off method (TTO) to obtain utility values of NSCLC patients in different treatment states in six countries or regions. We extracted the China-specific utility values, and the value of PFS was 0.804 while the value of PD was 0.321 in the base–case analysis. We also considered the disutility caused by adverse events (AEs) with a severity of grade ≥ 3 and an incidence of ≥5% (35). Because these AEs were expected to meaningfully reduce the quality of life, grade 1/2 AEs were generally self-limited (35). The AE incidence of iruplinalkib was provided by Qilu. Given that ALUR did not report the incidence of specific AEs of alectinib, the data were obtained from alectinib’s phase 2 trial (NP28673, (36)). The disutility values of AEs were collected from the published literature (34, 37). The one-time QALY decrements caused by AEs were calculated by combining disutility values with the incidence of AEs at the beginning of the first cycle. Details on the utility are shown in Table 2. Although compared with alectinib, iruplinalkib has less dosage frequency, the impact on utility caused by the dosage frequency is insignificant, for example, the study by Matza et al. (38) showed that utilities for all oral treatment regimens ranged from 0.80 (1 tablet) to 0.79 (7 tablets) in hepatitis C. There is no such study in the field of NSCLC; thus, utilities of the dosage frequency were not considered in this model.

**Table 2:** Key parameters and their variations.

Parameters	Value	Lower limit	Upper limit	Distribution	Data source
Utility values					
PFS	0.804	0.643	0.965	Beta	Nafees et al. (34)
PD	0.321	0.257	0.385	Beta	Nafees et al. (34)
Disutility of AEs					
Hypertension	−0.040	−0.048	−0.032	Beta	Nafees et al. (34)
Anemia	−0.073	−0.088	−0.058	Beta	Sivignon et al. (37)
Drug costs, per unit, $					
Iruplinalkib (60 mg)	20.48	16.38	24.58	Gamma	MENET database (39)
Alectinib (150 mg)	8.04	6.43	9.64	Gamma	MENET database (39)
Monitoring costs, per unit, $					
Outpatient	4.24	3.39	5.08	Gamma	Clinical opinion
Liver function test	14.12	11.30	16.95	Gamma	Clinical opinion
Cholesterol test	7.06	5.65	8.47	Gamma	Clinical opinion
Renal function test	10.88	8.70	13.05	Gamma	Clinical opinion
Electrocardiogram	4.24	3.39	5.08	Gamma	Clinical opinion
Electrolyte test	7.06	5.65	8.47	Gamma	Clinical opinion
Chest CT	68.50	54.80	82.20	Gamma	Clinical opinion
Serum creatine kinase test	6.36	5.08	7.63	Gamma	Clinical opinion
Subsequent therapy costs, per cycle, $					
Single-agent chemotherapy	1443.56	1154.85	1732.27	Gamma	Wu et al. (40) and adjusted by clinical opinion
Single-agent chemotherapy + bevacizumab	2957.50	2366.00	3549.00	Gamma	Wu et al. (40) and adjusted by clinical opinion
Anlotinib	580.93	464.75	697.12	Gamma	MENET database (39)
AEs cost, $					
Hypertension	14.45	13.01	15.90	Gamma	Gao et al. (41) and adjusted by clinical opinion
Anemia	595.79	536.21	655.37	Gamma	Gao et al. (41) and adjusted by clinical opinion
Incidence of AEs					
Hypertension-iruplinalkib	10.96%	——	——	——	Qilu
Hypertension-alectinib	0.68%	——	——	——	Ou et al. (36)
Anemia-iruplinalkib	0%	——	——	——	Qilu
Anemia-alectinib	5.07%	——	——	——	Ou et al. (36)

PFS, progression-free survival; PD, progression disease; AEs, adverse events; CT, computed tomography.

#### 2.2.3 Resource use and costs

Based on the perspective of China’s healthcare system, only direct costs, including drug, monitoring, subsequent therapy, and management of AEs, were considered (Table 2). Most costs were derived from the published literature, and on this basis, opinions of clinical experts were considered (including 46 clinical experts from more than 30 grade A tertiary hospitals in 4 provinces in China).

##### 2.2.3.1 Drug costs

The model calculated the drug cost in the PFS health state assuming that patients were treated until progression or death, according to the drug package inserts. Iruplinalkib and alectinib are both oral drugs and therefore, no administration costs were included. The recommended dosage of iruplinalkib is 60 mg once daily from day 1 to 7 and 180 mg once daily from day 8. The latest price (updated on 27 December 2023) of iruplinalkib was $20.48 (￥145)/60 mg, and the price of alectinib (600 mg twice daily) was $8.04 (￥56.90)/150 mg obtained from the MENET database (39).

##### 2.2.3.2 Monitoring costs

The items of monitoring were set based on the drug package inserts, and costs were calculated in both PFS and PD health states. The price data were collected by consulting clinical experts. Monitoring frequencies of iruplinalkib and alectinib were driven from drug package inserts and the CSCO Guidelines for NSCLC (2023) (details on the monitoring frequency are shown in Supplementary Table S4).

##### 2.2.3.3 Subsequent therapy costs

According to the CSCO Guidelines for NSCLC, single-agent chemotherapy, single-agent chemotherapy plus bevacizumab, and anlotinib were assumed as the posterior line treatment. The usage proportions and durations of the three therapies were driven by clinical experts (details are shown in Supplementary Table S5). One-time subsequent therapy costs were calculated based on the price, proportion, and duration and were included in the first cycle of PD health state.

##### 2.2.3.4 AEs costs

We considered the management caused by AEs with a severity of grade ≥ 3 and an incidence of ≥5% as they were expected to result in significant healthcare utilization (35). AE costs were calculated once in the first cycle. All costs are expressed in 2023 US dollars (1 USD = 7.08 CNY). Details of all cost parameters are shown in Table 2.

### 2.3 Sensitivity analyses

To address the uncertainty in the model, deterministic sensitivity analysis (DSA) and probabilistic sensitivity analysis (PSA) were performed. The DSA was conducted by varying one model input or assumption at a time. Ranges were based on 95% confidence intervals (CI) or varying the default input by ±20% (if 95% CIs were not applicable). The discount rate varied from 0 to 8% according to the China Guidelines for Pharmacoeconomics Evaluation 2020 (20). The PSA with 1,000 iterations was conducted to estimate the probability of iruplinalkib being cost-effective compared with alectinib using Monte-Carlo simulation. Uncertainty in the HRs of PFS and OS were estimated with normal distributions. Beta distributions were assigned for utilities of health states, and gamma distributions were assumed for costs. Cost-effectiveness acceptability curves (CEACs) were considered to show the probabilities of each arm being cost-effective at a wide range of WTP thresholds. Considering the long-term disease course of ALK-positive patients, we also performed a scenario analysis with a time horizon of 20 years and 30 years.

## 3 Result

### 3.1 Base case

The results of the base–case analysis are shown in Table 3. For iruplinalkib, the mean costs and QALYs were $61,278.53 and 2.77, respectively, while for alectinib, the mean costs and QALYs were $40,785.26 and 1.93, respectively. The incremental cost-effectiveness ratio (ICER) for iruplinalkib versus alectinib was $24,313.95/QALY.

**Table 3:** The results of base–case analysis.

Interventions	Iruplinalkib	Alectinib
Costs	$61,278.53	$40,785.26
Drug costs	$51,751.59	$31,858.55
Monitoring costs	$5,503.92	$3,826.43
Subsequent therapy costs	$4,017.36	$5,070.06
AEs costs	$5.66	$30.22
QALYs	2.77	1.93
LYs	5.16	3.97
Incremental costs	$20,493.27	——
Incremental QALYs	0.843	——
ICER	$24,313.95/QALY	——

AEs, adverse events; QALY, quality-adjusted life-year; LY, life year, ICER, incremental cost-effectiveness ratio.

### 3.2 Sensitivity analyses

**Figure 3:**
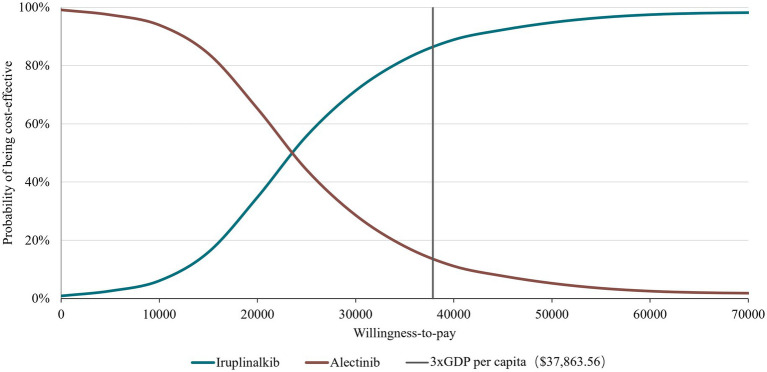
Cost effectiveness acceptability curve at different thresholds for willingness-to-pay. GDP, gross domestic product.

The results of the DSA are presented in Figure 2 with the top 10 parameters and illustrated that the results were robust with respect to changes in parameter inputs and most sensitive to changes in HRs and drug prices of iruplinalkib and alectinib. Probabilistic analysis showed an average QALY gain of 0.918 and incremental costs of $21,504.24, resulting in a probabilistic ICER of $23,417.46/QALY, which was consistent with the results of base–case analysis. The CEAC is shown in Figure 3, indicating that at a WTP threshold of three times China’s GDP *per capita* in 2023($37,863.56), the probability of iruplinalkib being cost-effective was nearly 90%. In the scenario analysis, when we varied the time horizon to 20 and 30 years, the ICERs were $25,022.80 and $23,887.17 per QALY, respectively. All ICERs were below the WTP, indicating that iruplinalkib was cost-effective.

## 4 Discussion

In recent years, innovative anticancer drugs have rapidly developed in China, and patients’ health has greatly improved. According to the Annual Drug Evaluation Report (2022) released by the Center for Drug Evaluation (CDE) of China (42), CDE approved 2019 investigational new drugs and 261 new drug applications for chemical and biological products, of which anticancer drugs accounted for the highest proportion, reaching 49.53 and 37.16%, respectively. However, the high price of anticancer drugs has become a heavy burden and raised public concerns about the long-term sustainability of patients and healthcare systems (43). China’s healthcare system, thus, attaches great importance to the value of innovative drugs, as well as patient affordability and accessibility. Evidence of economic evaluation was proposed for innovative drug pricing in many documents, referring to the adjustment of the NRDL (44).

Iruplinalkib was independently developed by a Chinese pharmaceutical company and has good efficacy. As the latest approved ALK-TKIs, it became a new treatment choice for ALK-positive NSCLC patients. Our study is the first evaluation that states the cost-effectiveness of iruplinalkib compared with alectinib in treating China’s ALK-positive crizotinib-resistant advanced NSCLC patients. Because there is no other economic evaluation for iruplinalkib due to its short time on the market. Given that there are no head-to-head clinical trials of iruplinalkib and alectinib, the unanchored MAIC is used in this study, which is recommended by NICE as the most appropriate indirect comparison method (4) to adjust the baseline characteristics of the population in the two clinical trials, to reduce survival data errors due to uneven distribution of covariates.

Based on the INTELLECT and ALUR study, and the updated drug prices, the analysis showed that ICERs for iruplinalkib versus alectinib were $25,022.80, $24,313.95, and $23,887.17 per QALY at 20, 25, and 30 years, respectively. The results of DSA showed that changes in HR for OS had the greatest influence on base–case ICER which might be due to the OS for iruplinalkib being immature, and changes in other parameters did not lead ICERs beyond the WTP threshold. The PSA results revealed that iruplinalkib had a 90% probability of being cost-effective, indicating that the base–case analysis results were robust. Considering its favorable clinical efficacy and safety, and the inhibitory activity against the ALK-resistant mutants including L1196M, C1156Y, and ALK-G1202R (16, 18), iruplinalkib is a cost-effective option for patients with ALK-positive crizotinib-resistant Advanced NSCLC in China.

There are several limitations in this study. First, there is no direct evidence comparing iruplinalkib with alectinib in this setting, so clinical data of alectinib were collected based on its international multicenter RCT (ALUR), of which patients were not limited to Chinese. Therefore, there may exist residual biases resulting from unobserved prognostic variables and effect modifiers (45), since the indirect comparing MAIC may be limited by the patients’ baseline information reported in the article. Second, because of the short median follow-up duration (18.2 months, 95%CI 16.8–18.8), a parametric survival model was used to extrapolate the long-term outcomes based on the immature OS curves. The results of the model may underestimate the efficacy of iruplinalkib and, thus, should be validated against long-term OS data from the trials or real-world evidence as the data become available. Third, some costs were absent, such as the monitoring cost; opinions derived from clinical experts were taken in the model which may lead to some biases. However, a series of sensitivity analyses indicated that model outcomes are robust since the main results remained unchanged in a wide range of parameter values.

In addition, it is important to notice that this result must be strictly considered within the Chinese setting, as the result of economic evaluation is closely related to health preferences, economic levels, and healthcare systems of different countries. When applying the results of this study to another healthcare setting, the suitability of data referring to the economy, clinic, humanism, and WTP threshold needs to be fully considered.

## 5 Conclusion

Based on available clinical trials (INTELLECT and ALUR), local resource utilization, and unit cost data, the present economic evaluation suggests that iruplinalkib was found to be cost-effective over alectinib in treating patients with ALK-positive crizotinib-resistant advanced NSCLC in China. Although the study is subjected to some uncertainties, the ICER appears to be modest with the WTP threshold for a high disease severity in this population.

## Data availability statement

The original contributions presented in the study are included in the article/Supplementary material, further inquiries can be directed to the corresponding authors.

## Ethics statement

This study was an economic evaluation analysis which based on previously publicly available data and does not involve any new studies of human or animal subjects performed by any of the authors.

## Author contributions

ZD: Data curation, Formal analysis, Funding acquisition, Methodology, Writing – original draft, Writing – review & editing. JX: Data curation, Formal analysis, Software, Writing – original draft, Writing – review & editing. FC: Data curation, Formal analysis, Supervision, Writing – review & editing. WZ: Data curation, Formal Analysis, Writing – review & editing. TR: Formal analysis, Writing – review & editing, Data curation. JQ: Data curation, Formal analysis, Writing – review & editing. YunL: Data curation, Formal analysis, Funding acquisition, Project administration, Writing – review & editing, Supervision. YuqL: Data curation, Formal analysis, Funding acquisition, Methodology, Writing – review & editing.
