# Supplementary material for: The cost-effectiveness of iruplinalkib versus alectinib in anaplastic lymphoma kinase-positive crizotinib-resistant advanced non-small-cell lung cancer patients in China
Source: Front Public Health. 2024 Apr 15;12:1333487. doi: 10.3389/fpubh.2024.1333487 (PMC11064164; doi:10.3389/fpubh.2024.1333487)
Supplement: Supplementary file 1 [file Data_Sheet_1.docx]

# Supplementary Material

## **Table 1 The result of Cox analysis**

| **Variables** | **Single-factor** | | **Multi-factor** | |
| --- | --- | --- | --- | --- |
|  | **HR** | **p** | **HR** | **p** |
| Age | 1.013 | 0.281 | 1.010 | 0.446 |
| Sex | 2.352 | **0.001**** | 2.713 | **0.000***** |
| ECOG PS | 1.604 | **0.074.** | 1.622 | **0.091.** |
| Histopathologic type | 0.532 | 0.532 | 0.335 | 0.323 |
| CNS metastasis at baseline | 0.600 | **0.045*** | 0.528 | **0.018*** |
| BOR to prior crizotinib | 1.072 | 0.535 | 1.003 | 0.984 |

“***”, p ＜ 0.001;“**”, p ＜ 0.01;“*”, p ＜ 0.05;“.”, p ＜0.1; ECOG, Eastern Cooperative Oncology Group; PS, performance status; CNS, central nervous system; BOR, best objective response.

## **Table 2 Matching variable selection of the unanchored MAIC**

| **Variables** | **In/Excluded** | **Reasons** |
| --- | --- | --- |
| Age | included | Published researches^[1][2][3][4][5]^took age as a matching variable in the unanchored MAIC |
| Sex | included | - Published researches^[1][2][3][4][5][6][7]^ took sex as a matching variable in the unanchored MAIC - COX analysis stated that sex as a prognostic variable significantly affected the efficacy |
| ECOG PS | included | - Published researches ^[1][2][3][4][5][6][7]^ took ECOG PS as a matching variable in the unanchored MAIC, and Nilsson et al.^[6]^、Smith et al.^[7]^ considered ECOG PS as a prognostic variable - COX analysis stated that ECOG PS as a prognostic variable significantly affected the efficacy |
| Histopathologic type | excluded | RCT of alectinib (ALUR) did not report information of histopathologic type |
| CNS metastasis at baseline | included | - Published researches ^[1][2][3][4][6][7]^ took CNS metastasis at baseline as a matching variable in the unanchored MAIC, and Reckamp et al.^[1]^ considered CNS metastasis at baseline as a prognostic variable - COX analysis stated that CNS metastasis at baseline as a prognostic variable significantly affected the efficacy |
| BOR to prior crizotinib | excluded | RCT of alectinib (ALUR) did not report information of BOR to prior crizotinib |

## **Table 3 Parametric functions fitting for PFS and OS of iruplinalkib**

| **Model** | **PFS** | | **OS** | |
| --- | --- | --- | --- | --- |
|  | **AIC** | **BIC** | **AIC** | **BIC** |
| Exponential | 646.9848 | 649.9684 | 627.2898 | 630.2734 |
| Weibull | 637.3656 | 643.3328 | 618.9375 | 624.9047 |
| Gompertz | 643.7625 | 649.7297 | 625.4652 | 631.4324 |
| Log-normal | 632.2385 | 638.2057 | 612.0247 | 617.9919 |
| Log-logistic | 634.4127 | 640.3799 | 615.7738 | 621.741 |
| Generalized Gamma | 634.2140 | 643.1649 | 610.7790 | 619.7298 |

## **Figure 1 KM and parametric survival curve fits for PFS of iruplinalkib**


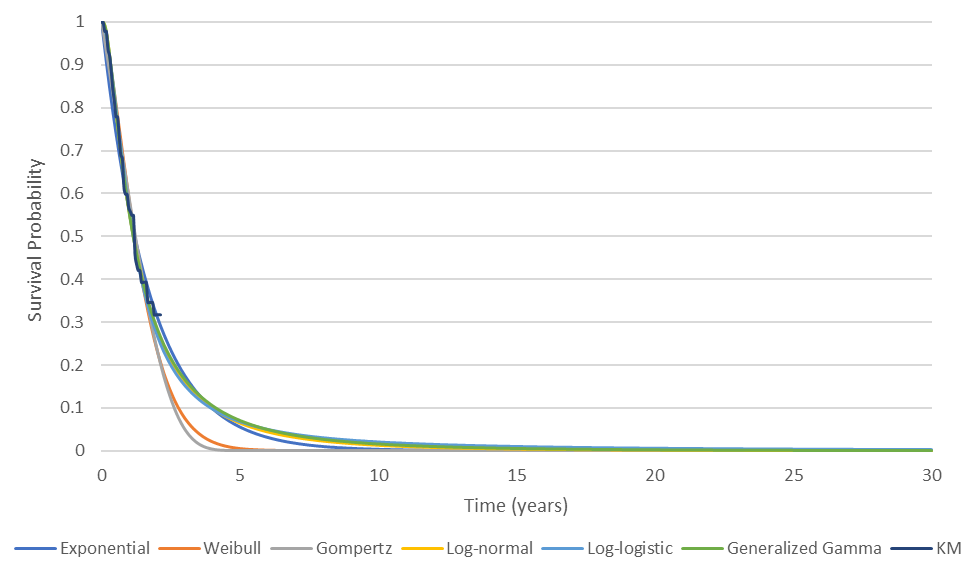


## **Figure 2 KM and parametric survival curve fits for OS of iruplinalkib**


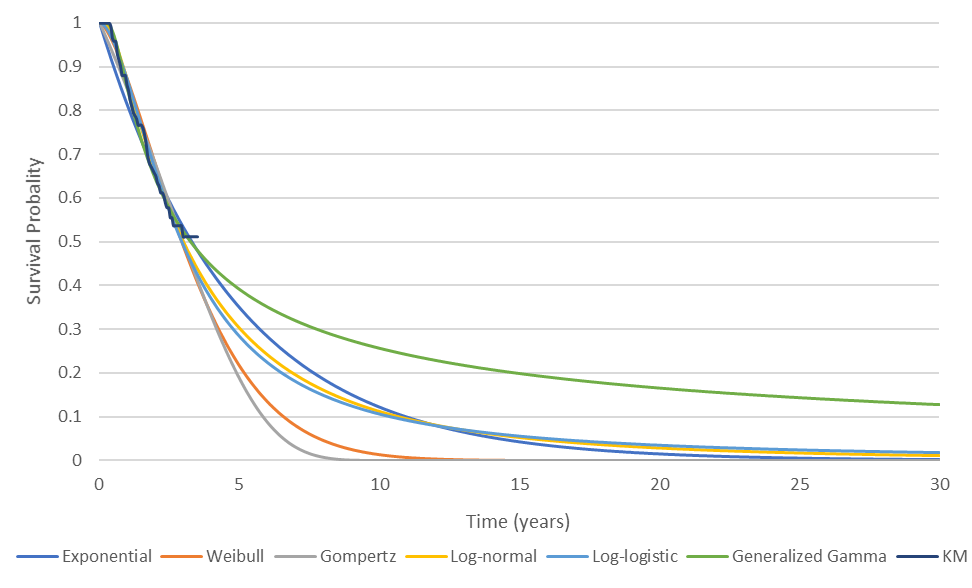


## **Table 4 Monitoring frequency of iruplinalkib and alectinib**

|  | **iruplinalkib** | **alectinib** |
| --- | --- | --- |
| Outpatient | Once every 6 weeks | Once every 6 weeks |
| Liver function test | Once every 4 weeks | Once every 4 weeks |
| Cholesterol test | Once every 6 weeks | N/A |
| Renal function test | Once every 6 weeks | Once every 6 weeks |
| Electrocardiogram | Once every 6 weeks | N/A |
| Electrolyte test | Once every 6 weeks | N/A |
| Chest CT | Once every 6 weeks | Once every 6 weeks |
| Serum creatine kinase test | N/A | Once every 6 weeks |

N/A: Not Applicable.

## **Table 5 Usage proportions and durations of subsequent therapies**

|  | **Proportions** | **Durations** | **Data source** |
| --- | --- | --- | --- |
| single-agent chemotherapy | 22.22% | 12 weeks | Clinical opinion |
| single-agent chemotherapy + bevacizumab | 55.56% | 12 weeks | Clinical opinion |
| anlotinib | 22.22% | 7 weeks | Clinical opinion |

# References

1. Reckamp KL, Lin HM, Cranmer H, Wu Y, Zhang P, Kay S, et al. Overall survival indirect treatment comparison between brigatinib and alectinib for the treatment of front-line anaplastic lymphoma kinase-positive non-small cell lung cancer using data from ALEX and final results from ALTA-1L. Curr Med Res Opin. (2022)38(9):1587-1593. doi:10.1080/03007995.2022.2100653
2. Reckamp KL, Lin HM, Cranmer H, Wu Y, Zhang P, Walton LJ, et al. Indirect comparisons of brigatinib and alectinib for front-line ALK-positive non-small-cell lung cancer. Future Oncol. (2022)18(20):2499-2510. doi: 10.2217/fon-2022-0194
3. Reckamp K, Lin HM, Huang J, Proskorovsky I, Reichmann W, Krotneva S, et al. Comparative efficacy of brigatinib versus ceritinib and alectinib in patients with crizotinib-refractory anaplastic lymphoma kinase-positive non-small cell lung cancer. Curr Med Res Opin. (2019)35(4):569-576. doi: 10.1080/03007995.2018.1520696
4. Li J, Knoll S, Bocharova I, Tang W, Signorovitch J. Comparative efficacy of first-line ceritinib and crizotinib in advanced or metastatic anaplastic lymphoma kinase-positive non-small cell lung cancer: an adjusted indirect comparison with external controls. Curr Med Res Opin. (2019)35(1):105-111. doi: 10.1080/03007995.2018.1541443
5. Tan DS, Araújo A, Zhang J, Signorovitch J, Zhou ZY, Cai X, et al. Comparative Efficacy of Ceritinib and Crizotinib as Initial ALK-Targeted Therapies in Previously Treated Advanced NSCLC: An Adjusted Comparison with External Controls. J Thorac Oncol. (2016)11(9):1550-1557. doi: 10.1016/j.jtho.2016.05.029
6. Nilsson FOL, Asanin ST, Masters ET, Iadeluca L, Almond C, Cooper M, et al. The Cost-Effectiveness of Lorlatinib Versus Chemotherapy as a Second- or Third-Line Treatment in Anaplastic Lymphoma Kinase (ALK)-Positive Non-small-cell Lung Cancer in Sweden. Pharmacoeconomics. (2021)39(8):941-952. doi: 10.1007/s40273-021-01015-8
7. Smith S, Albuquerque de Almeida F, Inês M, Iadeluca L, Cooper M. Matching-Adjusted Indirect Comparisons of Lorlatinib Versus Chemotherapy for Patients With Second-Line or Later Anaplastic Lymphoma Kinase-Positive Non-Small Cell Lung Cancer. Value Health. (2023)26(1):64-70. doi: 10.1016/j.jval.2022.07.002
